# Diagnostic Potential of Periostin, Galectin-3 and Tenascin C Serum Measurements in Inflammatory Bowel Disease: Pilot Study

**DOI:** 10.3390/ijms262110439

**Published:** 2025-10-27

**Authors:** Aleksandra Górecka, Agnieszka Jura-Półtorak, Anna Szeremeta, Katarzyna Komosinska-Vassev

**Affiliations:** Department of Clinical Chemistry and Laboratory Diagnostics, Faculty of Pharmaceutical Sciences in Sosnowiec, Medical University of Silesia in Katowice, 41-200 Sosnowiec, Poland

**Keywords:** inflammatory bowel disease, ulcerative colitis, Crohn’s disease, periostin, galectin-3, tenascin C, extracellular matrix, biomarker

## Abstract

Inflammatory bowel diseases (IBD), encompassing ulcerative colitis (UC) and Crohn’s disease (CD), are characterized by a complex interplay between chronic inflammatory process and extracellular matrix (ECM) remodeling. This pilot study aims to evaluate the serum levels of three ECM-related proteins—periostin, galectin-3, and tenascin C—as biomarkers supporting IBD diagnosis. Serum concentration of periostin, galectin-3 and tenascin C were measured using the ELISA method in 49 patients with IBD and 30 healthy individuals. Periostin and galectin-3 levels differed significantly between IBD patients and healthy individuals, whereas tenascin C levels did not show a significant difference. The ROC curve analysis identified periostin as the most promising biomarker for differentiation of both UC and CD from healthy individuals. In UC patients, periostin distinguished them from healthy individuals with excellent accuracy (AUC = 0.922), high sensitivity (100%), and good specificity (77.8%). Similarly, in CD patients, periostin demonstrated excellent diagnostic performance with AUC of 0.943, high sensitivity (100%) and good specificity (77.8%). Galectin-3 also showed potential as a diagnostic marker, which discriminated both UC and CD patients with high accuracy of AUC = 0.745 in UC and AUC = 0.691 in CD groups. Moreover, serum galectin-3 levels correlated with CRP levels (r = 0.603, *p* < 0.05) in the CD group. After one year of conventional anti-inflammatory treatment in CD patients, levels of periostin (*p* < 0.001) and galectin-3 (*p* < 0.05) significantly decreased. In contrast UC patients, receiving anti-TNF-α biological therapy showed a significant increase in galectin-3 (*p* < 0.05) concentrations. Obtained results indicate that circulating periostin and galectin-3 emerge as promising biomarkers for differentiating both UC and CD patients from healthy individuals. Given the significant correlation between galectin-3 and CRP serum levels, galectin-3 may also serve as a useful marker for monitoring disease activity in CD. Furthermore, galectin-3 may be helpful in monitoring the response to both biological or conventional anti-inflammatory treatment in UC and CD patients. Periostin, in turn, may be particularly valuable for evaluating efficacy of conventional anti-inflammatory therapy in CD.

## 1. Introduction

Inflammatory bowel disease (IBD) is a group of chronic, inflammatory disorders of the gastrointestinal tract, primarily encompassing ulcerative colitis (UC) and Crohn’s disease (CD). The pathogenesis of IBD is multifactorial, involving an abnormal immune response, loss of intestinal barrier integrity, intestinal dysbiosis, genetic predisposition and environmental factors. The course of IBD is characterized by alternating flares and remissions of symptoms including diarrhea, rectal bleeding, abdominal pain, weight loss and fatigue. Despite similar features, UC and CD differ significantly in terms of disease location, extraintestinal manifestations and risk of fistula formation [1,2,3,4]. Regarding chronicity and the clinically burdensome course of IBD, accurate and early diagnosis is extremely important. IBD diagnosis is usually based on endoscopic examinations, clinical symptoms and laboratory biomarkers. Endoscopic examination provides important clinical value; however, it is also a highly invasive diagnostic method. Moreover, endoscopic methods are sometimes not sufficient on their own to make an accurate diagnosis. Therefore, diagnosis of IBD is usually supported by laboratory methods including assessment of fecal calprotectin, which is used for early IBD diagnosis and monitoring of disease activity. Although fecal calprotectin is a sensitive biomarker, it lacks specificity for IBD. Increased fecal calprotectin concentrations may be observed in gastrointestinal infections, celiac disease or colorectal cancer [5,6]. Therefore, there is a serious need to identify new IBD biomarkers that could further support—with high sensitivity and specificity—not only diagnosis of IBD, but also subsequent differentiation of patients to UC or CD. Additionally, during IBD the chronic inflammatory state results in abnormal remodeling of the extracellular matrix (ECM). This disruption of ECM remodeling impairs intestinal healing and may result in development of fibrosis, strictures and/or fistulas [7]. In spite of these complications, biomarkers used in evaluating disease activity and response to implemented treatment mostly encompasses measurements of fecal calprotectin and serum CRP levels, which reflect intestinal inflammation, rather than intestinal fibrosis and proper healing. Identification of these new biomarkers could limit the necessity of endoscopic and imagining examinations. Additionally, these biomarkers could enable an early detection of strictures and fibrosis, thus ensuring non-invasive and precise monitoring of IBD.

Among ECM components and fibrosis-associated markers implicated in IBD periostin, galectin-3 and tenascin C are especially relevant. These three ECM-related proteins were selected based on their central involvement in key pathophysiological processes of IBD, particularly chronic inflammation, ECM remodeling, and intestinal fibrosis. These mechanisms underly disease progression and complications such as strictures and fistulas. Moreover, their association with inflammation and fibrotic processes has been demonstrated in other chronic inflammatory diseases, including asthma, pulmonary fibrosis, rheumatoid arthritis, and chronic kidney disease [8,9,10,11]. An additional important factor in their selection was the availability of well-standardized, commercially available immunoenzymatic assays (ELISA kits), allowing for reproducible, reliable, and non-invasive quantification in serum samples. These methodological advantages make these markers particularly suitable for clinical use and potential diagnostic applications.

First of the analyzed biomarkers is periostin, a pleiotropic matricellular protein. Periostin is engaged in ECM organization and contributes to the fibrosis process by stabilizing components like collagens and elastin. It also enhances inflammatory response by activating NF-κB pathway. As such, periostin may serve as a biomarker of both inflammation and early fibrogenesis in IBD [8,12,13,14,15].

Tenascin C is another ECM protein involved in wound healing, inflammation, and fistula remodeling. Its expression is typically low in adults; however, it may be upregulated in stem cell niches and tendons, as well as under pathological conditions such as chronic inflammation and/or tissue injury. Elevated expression of tenascin C in fibrotic and inflamed tissue supports its potential as a biomarker of chronic inflammation and advanced fibrosis in IBD [9,16,17,18].

Galectin-3 plays a crucial role in both immune response and fibrosis. It regulates the activation of fibroblast, proliferation of mesenchymal cells, and synthesis of ECM components (such as collagens), which may promote fibrotic remodeling. Galectin-3 is markedly overexpressed during inflammation, as it acts as a damage-associated molecular pattern (DAMP). Therefore, as it enhances the recruitment of neutrophils and macrophages into sites of inflammation, galectin-3 can amplify the inflammatory cascade and tissue injury [10,11,19,20,21,22,23].

Taken together, the selection of periostin, tenascin C and galectin-3 was based on both biological plausibility and technical feasibility. Their serum concentrations may reflect mucosal inflammation and fibrotic remodeling in IBD. Therefore, the aim of this study was to evaluate the utility of periostin, tenascin C and galectin-3 serum profiles for supporting the early diagnosis, differentiation between UC and CD, and monitoring of treatment response.

## 2. Results

### 2.1. Characteristics of Patients

This study included 49 patients diagnosed with inflammatory bowel disease, comprising 31 with ulcerative colitis and 18 with Crohn’s disease, whose clinical characteristics are presented in Table 1. The UC group consisted of 13 females and 18 males, with a mean age of 33 years. In this group, 58.1% of patients had severe disease, defined as a score of 3 on the Mayo endoscopic scale (MES), while 42% had moderate disease (MES = 2). No patients in the UC group presented mild disease or clinical remission. The median CRP level among UC patients was 3.37 mg/L, with 42% of patients exhibiting a normal CRP level (defined as <0.05 mg/L), suggesting low or absent systemic inflammation. After one year of biological treatment, UC patients presented a statistically significant decrease in disease activity (*p* < 0.005), with a median MES score of 2. In the post-treatment group, severe disease (MES = 3) was observed in 32.3% of patients, moderate disease (MES = 2) in 29%, mild (MES = 1) in 35.5%, and clinical remission (MES = 0) in 3.2% of patients. Additionally, a minor, however not statistically significant, decrease in CRP levels was observed following treatment, with a post-treatment median of CRP of 2.41 mg/L. A similar trend was noted for serum calprotectin levels, which decreased slightly from 2782.9 to 2708.3 ng/mL after treatment.

The CD group included 8 females and 10 males with a mean age of 32 years. According to the Montreal classification, regarding disease location, L1 (ileal) category was observed in five patients, L2 (colonic) in seven patients, L3 (ileocolonic) in seven patients and L4 (isolated upper disease) in one patient. Most patients with Crohn’s disease (94.4%) presented moderate disease, while only 5.6% had severe disease. The median CRP level in this group was 13.9 mg/L, and 33.3% of patients had normal CRP concentrations. Although CRP levels were generally higher in CD patients compared to those with UC, the difference was not statistically significant. After one year of anti-inflammatory treatment, the median disease activity was 270.8 points in CDAI scale. However, this reduction in disease activity was not statistically significant. After treatment, no patients were diagnosed with severe disease; 88.9% had moderate disease, and 11.1% demonstrated mild disease. In the post-treatment CD group, CRP levels slightly increased, while serum calprotectin concentrations decreased from a mean 3537.52 to 2915.3 ng/mL. These changes in CRP and calprotectin levels were not statistically significant.

### 2.2. Serum Profile of Periostin, Galectin-3 and Tenascin C in Patients with Inflammatory Bowel Disease and Healthy Individuals

**Figure 1:**
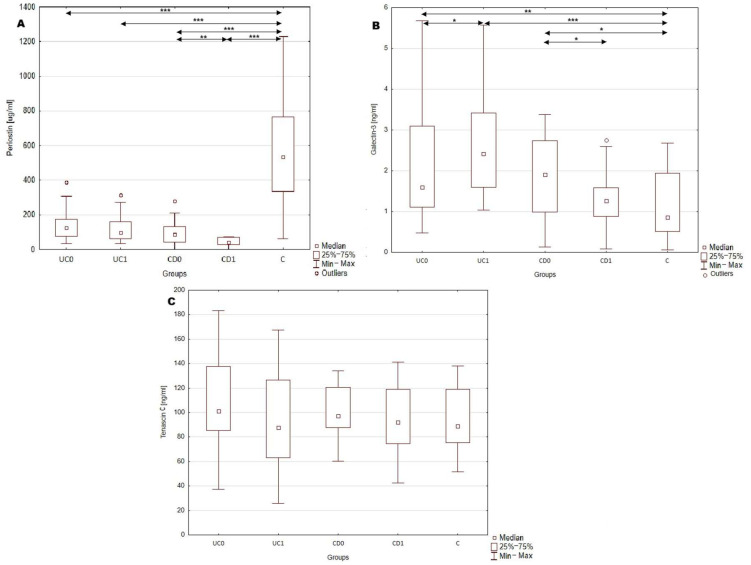
Serum profile of periostin, galectin-3 and tenascin C in serum of IBD patients. (**A**), serum periostin concentration in analyzed groups; (**B**), serum galectin-3 concentration in analyzed groups; *, *p* < 0.05; **, *p* < 0.001; ***, *p* < 0.0001; (**C**), serum tenascin C concentration in analyzed groups. C, control group; CD0, patients with Crohn’s disease before treatment; CD1, patients with Crohn’s disease after year of anti-inflammatory treatment; UC0, patients with ulcerative colitis before treatment; UC1, patients with ulcerative colitis after a year of biological treatment.

In this study serum concentration of periostin, galectin-3 and tenascin C were measured in patients with IBD and healthy individuals. The results are presented in Table 2 and Figure 1. The most significant difference in serum profile of the analyzed biomarkers between IBD patients and healthy individuals was noted in the case of periostin. The concentration of this biomarker was nearly five times lower in UC patients compared to healthy individuals (*p* < 0.0001; 119.33 vs. 548.8 μg/mL). Even greater differences were noted in the CD group, where the serum periostin concentrations showed an almost six-fold decrease compared to the control group (*p* < 0.0001; 99.45 vs. 548.8 μg/mL). Considering the disease location, periostin concentration was 127.05 ± 76.1 μg/mL in patients with L1 (ileal); 72.6 ± 48.0 μg/mL in L2 (colonic); 107.33 ± 65.1 μg/mL in L3 (ileocolonic) and 130.3 μg/mL in L4 (isolated gastrointestinal tract) phenotype of CD patients. A statistically significant difference between IBD patients and healthy individuals was also noted in galectin-3 levels. In the group of patients with UC, galectin-3 concentration was increased by 71.6% with regard to healthy individuals (*p* < 0.0005; 1.63 vs. 0.95 ng/mL). A similar increase was observed in CD group, where galectin-3 levels were 102.1% higher than in the control group (*p* < 0.05; 1.92 vs. 0.95 ng/mL). Galectin-3 concentration was 0.94 ± 0.4 ng/mL in L1; 1.88 ± 0.5 ng/mL in L2; 2.23 ± 1.2 ng/mL in L3 and 2.97 ng/mL in L4 patients with CD. No significant differences were observed in serum tenascin C levels (including both UC and CD) and healthy individuals. Concentration of tenascin C was 102.6 ± 28.2 ng/mL in L1; 102.09 ± 19.8 ng/mL in L2; 96.1 ± 26.6 ng/mL in L3 and in 102.6 ng/mL L4 patients with CD. Moreover, the serum profile of periostin, galectin-3 and tenascin C did not differ significantly between UC and CD patients.

### 2.3. Evaluation of Serum Periostin, Galectin-3 and Tenascin C Profile as Diagnostic Biomarkers of Ulcerative Colitis and Crohn’s Disease

**Figure 2:**
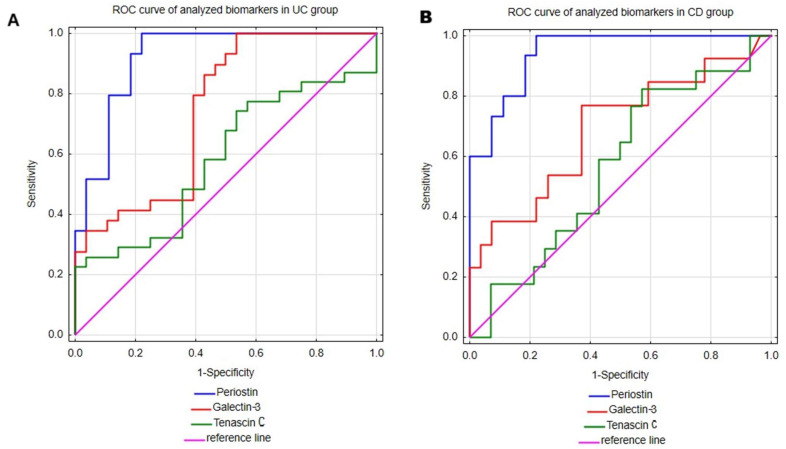
The ROC curve analyses of periostin, galectin-3 and tenascin C were performed in patients with ulcerative colitis (**A**) and Crohn’s disease (**B**).

The main aim of our study was to assess the utility of serum measurements of periostin, galectin-3 and tenascin C in the diagnosis of UC and CD. Therefore, receiver operating characteristic (ROC) curve analysis was performed to evaluate the ability of mentioned biomarkers to distinguish UC or CD patients from healthy individuals. The results are presented in Table 3 and Figure 2. Among all the analyzed parameters periostin emerged as the most promising biomarker for both UC and CD. In UC patients, the serum periostin profile enabled discrimination from healthy individuals with excellent accuracy, yielding an area under the curve (AUC) of 0.922 (95% CI; 0.850–0.995). Moreover, the test was characterized by high sensitivity (100%) and great specificity (77.8%), together with excellent positive predictive value (PPV; 82.8%) and negative predictive value (NPV; 100%). Taking into consideration superior sensitivity and NPV in comparison to specificity and PPV, this test may be more prone to overdiagnosing UC. Similarly, in the CD group, periostin measurements allowed accurate differentiation of CD patients from healthy individuals, with AUC 0.943 (95% CI; 0.88–1). The serum periostin profile in CD exhibited similarly high values of diagnostic performance indicators as in the UC group, with comparable values of sensitivity (100%), specificity (77.8%) and NPV (100%), but a slightly lower value of PPV (71.4%).

Galectin-3 measurements in serum also proved useful in differentiating both UC and CD patients from healthy individuals. This test demonstrated a great ability to differentiate UC patients from healthy individuals with AUC 0.745 (95% CI; 0.616–0.875). Additionally, galectin-3 assessments presented excellent sensitivity (100%), but low specificity (46.4%), indicating a high risk of false-positive UC diagnoses. In the group of patients with CD, galec-tin-3 demonstrated lower diagnostic accuracy metrics compared to the UC group, with an AUC of 0.691 (95% CI; 0.506–0.876). Among CD patients, this test presented good sensitivity (76.9%) and satisfactory specificity (63%). However, due to the relatively low PPV (reaching only 50%), the diagnostic utility of galectin-3 in CD appears to be limited.

The last analyzed biomarker was tenascin C, which demonstrated only moderate ability to differentiate both UC and CD patients from healthy individuals with AUCs of 0.582 (95% CI; 0.434–0.73) and 0.567 (95% CI; 0.395–0.739), respectively. In the UC group, tenascin C demonstrated excellent specificity (100%), but poor sensitivity (22.6%), indicating limited utility of this biomarker in supporting the diagnosis of UC. In CD patients, tenascin C showed high sensitivity (82.4%), but unsatisfactory specificity (42.9%), which similarly limits its diagnostic usefulness in CD.

### 2.4. Utility of Serum Periostin, Galectin-3 and Tenascin C in Assessing Disease Activity in Inflammatory Bowel Disease

This study analyzed the potential role of serum periostin, galectin-3, and tenascin-C in assessing disease activity among IBD patients. The only significant, positive correlation was observed between galectin-3 and CRP (r = 0.603, *p* < 0.05) concentrations in patients with Crohn’s disease, suggesting a potential link between galectin-3 and systemic inflammatory response in this subgroup. Neither periostin nor tenascin C level correlated with CRP level in all IBD patients. Moreover, no valid relationship was observed in the case of the analyzed biomarkers and disease activity in both UC and CD patients.

### 2.5. Effect of Implemented Treatment on the Serum Profile of Periostin, Galectin-3 and Tenascin C in Patients with IBD

Serum concentrations of periostin, galectin-3 and tenascin C were measured before and after one year of treatment in order to assess the impact of implemented therapy on the profile of analyzed biomarkers. Patients with ulcerative colitis received biological anti-TNF-α treatment with adalimumab, which led to an improvement in average disease activity in this group. Following adalimumab therapy, serum galectin-3 levels significantly increased by 49.1% (*p* < 0.05; 1.63 vs. 2.42 ng/mL). However, no other significant differences in serum periostin or tenascin C levels were observed in this group of patients. In the CD group conventional anti-inflammatory treatment was implemented, which did not result in a statistically significant change in average disease activity. Nevertheless, after one year of therapy CD patients showed a significant decrease in serum periostin level by 57.8% (*p* < 0.005; 98.03 vs. 41.38 μg/mL), and galectin-3 levels by 30.7% (*p* < 0.05; 1.92 vs. 1.33 ng/mL). No significant changes were observed in serum tenascin C concentrations in the CD group following treatment.

## 3. Discussion

### 3.1. Serum Profile of Periostin, Galectin-3 and Tenascin C in Patients with Inflammatory Bowel Disease and Healthy Individuals

In this study, serum profiles of periostin, galectin-3 and tenascin C were analyzed to evaluate their potential as new, non-invasive biomarkers supporting IBD diagnosis. Among the analyzed biomarkers, significant differences in serum concentrations of periostin and galectin-3 were observed between IBD patients and healthy individuals. Serum periostin levels were decreased in both UC and CD patients compared to healthy individuals. This observed down-regulation of periostin may be related to disease activity. Periostin expression is known to increase during the proliferative and remodeling phases of tissue repair, but not during the inflammatory phase. In our study, the pre-treatment group of UC patients consisted predominantly of patients with severe disease, and none were in clinical remission. Therefore, the reduced periostin level may reflect a prolonged inflammatory state in the intestinal tissue of patients with active disease [24]. The CD group also consisted of patients with active disease being evaluated as severe or moderate. Similarly to the UC group, patients with CD presented decreased concentration of periostin. This hypothesis is supported by the study of Coelho et al. [25] conducted in a pediatric population of IBD patients. The researchers noted that individuals with active disease exhibited significantly lower circulating periostin levels compared to those in clinical remission. Consistent with our findings, Coelho et al. also observed lower periostin concentrations in active IBD patients compared to healthy controls, although in their study that difference was not statistically significant. Thus, down-regulation of circulating periostin during active disease may represent a shared feature across both pediatric and adult populations of IBD patients. Reduced periostin expression may result from sustained inflammation, which impairs subsequent proliferative and remodeling phases of mucosal healing. In fact, in a cutaneous wound model, periostin deficiency led to reduced wound contraction due to impaired migration of myofibroblasts and delayed re-epithelization associated with diminished proliferation of keratinocytes [26]. Thus, during IBD, the observed decrease in serum periostin levels may disrupt and prolong the healing of the intestinal mucosa. The underlying cause of decreased circulating periostin in IBD remains unclear. However, it may be related to diminished release into the bloodstream resulting from enhanced periostin deposition in intestinal tissue. This hypothesis is supported by the study of Koh et al. [15], which demonstrated up-regulated expression of periostin in colonic tissue of UC patients compared to healthy individuals. Increased intestinal expression of periostin during IBD may be related to enhanced epithelial-to-mesenchymal transition, fibroblasts proliferation, and collagen synthesis, all of which contribute to the development of intestinal fibrosis. Moreover, TGF-β is known to stimulate periostin expression and periostin itself may promote further TGF-β production, potentially creating a self-reinforcing fibrotic loop [12]. Thus, during IBD, periostin accumulation within the intestinal tissue may lead to increased extracellular matrix (ECM) stiffness, formation of strictures, and progressive loss of intestinal function. Unfortunately, in the study by Koh et al., periostin concentration in serum was not measured, nor were disease activity or duration reported. In contrast, Takedomi et al. [27] reported elevated colonic and serum periostin expression in UC patients compared to controls, suggesting that serum periostin concentrations may reflect its intestinal tissue expression. It should be noted, however, that colonic biopsies in Takedomi et al.’s study were collected from only six UC patients and three individuals with early-stage sigmoid colon cancer, who served as a control group. Although the increase in periostin expression in the intestinal tissue of UC patients was statistically significant, three out of six UC patients demonstrated periostin expression comparable to controls. Moreover, serum periostin concentration was assessed in 111 UC patients and only 13 healthy individuals, with a heterogeneous distribution in the UC group—31.5% of patients demonstrated periostin levels similar to the mean value in the control group. Notably, the UC patients in Takedomi et al.’s study were predominantly in remission or had mild disease (Mayo endoscopic score of 0 or 1), whereas our cohort included only patients with moderate or severe disease. These differences in disease activity, the limited size of the control group, and the inclusion of patients with sigmoid colon cancer rather than entirely healthy individuals as controls, make direct comparisons between the studies challenging. The relationship between tissue and circulating periostin levels in UC remains to be fully elucidated. Therefore, it remains unclear whether serum and tissue levels of periostin correlate directly, depending on the phase of wound healing, or demonstrate an inverse relationship due to its accumulation in intestinal tissue.

The main objective of our study, however, was to assess the diagnostic utility of periostin measurements in IBD. The clinical utility of periostin is also being investigated in other inflammatory diseases, including chronic kidney disease, asthma and rheumatoid arthritis. Despite the encouraging results, periostin assessments are not yet implemented in clinical practice [8]. Indeed, in our study periostin occurred as the most promising biomarker, with comparable diagnostic performance in both UC and CD group of patients. Periostin measurements demonstrated excellent ability to differentiate UC and CD patients from healthy individuals, with 100% of sensitivity and high specificity in both groups. Additionally, periostin assessment in UC and CD were associated with low incidence of false negative results (i.e., underdiagnoses), although with a tendency toward overdiagnosis. To reduce the risk of false positive UC and CD diagnoses, periostin measurements could be combined with another biomarker characterized by higher specificity. Considering the widespread clinical use of fecal calprotectin in IBD diagnostics, future studies may explore the combined assessment of serum periostin and fecal calprotectin. Such a biomarker panel could enhance the accuracy of IBD detection, particularly in distinguishing affected patients from healthy individuals. Given the known limitations of fecal calprotectin, this approach should also be evaluated in individuals with other gastrointestinal disorders included as a second control group. Results of this study indicate that periostin alone demonstrated potential as a useful biomarker in the IBD diagnostic process.

Our study demonstrated an increased serum level of galectin-3 in both UC and CD patients compared to healthy individuals. Similar findings were reported in the studies by Yu et al. [28] and Frol’ova et al. [29], in which elevated galectin-3 levels were also observed in the serum of both UC and CD patients relative to healthy controls. Additionally, Frol’ova et al. noted that the upregulation of serum galectin-3 occurs not only in active disease but also during clinical remission. The researchers further observed that galectin-3 expression in intestinal tissue of IBD patients was prominent in CD14+ cells (such as monocytes and macrophages), but weak or absent in enterocytes. In contrast, colonic samples from non-IBD individuals showed predominant galectin-3 expression in enterocytes, with little to no expression in CD14+ cells. These findings not only stay in line with our results, but also indicate potential cellular source of galectin-3 in IBD. Under physiological conditions, galectin-3 is synthesized and stored in cytoplasm. However, it can be secreted by activated immune cells or passively released from damaged cells upon microbial or inflammatory stimuli. Once released, galectin-3 modulates the inflammatory response, indicating that its enhanced expression in IBD is not only a consequence of inflammation but may also contribute to disease progression [20]. Notably, Markovic et al. [21] proposed that galectin-3 may interact with glycans of bacterial lipopolysaccharides (LPS). Galectin-3 may promote the breakdown of LPS aggregates and enhance their interaction with CD14, thereby facilitating TLR4 activation. TLR4 activation subsequently leads to upregulation of the NF-κB signaling pathway and increased synthesis of proinflammatory cytokines such as TNF-α and IL-6, as well as activation of the NLRP3 inflammasome complex, further amplifying inflammatory cascade [30]. The proinflammatory role of galectin-3 was confirmed in the study of Marokvic et al. [21] using an animal model, in which galectin-3 enhanced NLRP3 inflammasome activation and of IL-1β synthesis in macrophages, ultimately leading to acute colitis. Given that galectin-3 upregulation contributes to the amplification of the inflammatory response, its measurement in serum may serve as a marker of intestinal inflammation during IBD.

Interestingly, considering the role of galectin-3 in inflammatory and fibrotic processes, the U.S. Food and Drug Administration (FDA) has approved galectin-3 measurements as a prognostic biomarker in chronic heart failure. Moreover, the clinical utility of galectin-3 assessments is being explored in other chronic inflammatory conditions, such as chronic kidney disease, rheumatoid arthritis, systemic sclerosis and liver fibrosis [31,32]. Indeed, in our study serum galectin-3 levels enabled us to differentiate patients with UC from healthy individuals with 100% of sensitivity and NPV, although unsatisfactory specificity. These results indicate strong identification of UC patients and a low risk of false negative diagnoses, but a considerable risk of false positive classification. In the CD group, galectin-3 assessments also presented moderate diagnostic utility in differentiating CD patients from healthy controls. The test showed moderate sensitivity and NPV, but low PPV, reflecting a suboptimal ratio of true positive to false positive results. Considering the overall value of diagnostic indicators in both UC and CD groups, serum galectin-3 measurement appears to be more valuable in diagnosis of UC than CD. In the analyzed groups, the differences in galectin-3 levels were less pronounced compared to those observed for periostin, resulting in lower statistical power. This highlights the need to include a larger number of patients to strengthen the analysis. Therefore, these results should be further validated in larger, more phenotype-diverse IBD cohort. Considering the inflammatory nature of IBD and established association of galectin-3 with different pathological conditions including chronic heart failure, further studies should also include a second control group. This additional control group could comprise patients with other chronic inflammatory disorders to better evaluate the diagnostic performance of the assessed biomarkers.

This study revealed that circulating tenascin C concentrations did not differ between IBD patients and healthy individuals. Our findings are in contrast to those reported by Ning et al. [33], who observed increased serum levels of tenascin C in both UC and CD patients compared to healthy individuals. Importantly, the discrepancies between our results and those of Ning et al. do not appear to be attributable to differences in disease activity, as both studies noted elevated levels of tenascin C during active disease phases. A more likely explanation lies in methodological differences. Tenascin C is present in circulation in various isoforms arising from post-transcriptional and post-translational modifications [9]. This may influence the analytical specificity of different assays. In the study of Ning et al., tenascin C levels were measured with the Human Tenascin C Large (FNIII-0C) kit (Immuno-Biological Laboratories Co., Gunma, Japan), while our study used the Tenascin C ELISA kit from Cloud-Clone Corporation (Houston, TX, USA). Our study evaluated the total concentration of circulating tenascin C, while Ning et al. measured only the large isoform. This large isoform of tenascin C is known to be associated with inflammation and tissue injuries [9,33]. These findings suggest that although total circulating tenascin C may not differ in IBD patients compared to healthy individuals, specific pro-inflammatory isoforms may be elevated. Therefore, diagnostic assessment of isoform-specific tenascin C variants rather than its total level may provide more clinically relevant information in IBD.

### 3.2. Utility of Serum Periostin, Galectin-3 and Tenascin C Profile in Evaluating Disease Activity and Response to Treatment in Patients with IBD

The utility of periostin, galectin-3 and tenascin C measurements was evaluated not only in IBD diagnosis, but also in monitoring disease activity. Therefore, the relationship between these biomarkers and disease activity indices and CRP levels were assessed. While none of the biomarkers presented a statistically significant correlation with disease activity scores in IBD patients, a notable association (r = 0.603, *p* < 0.05) was observed between galectin-3 and CRP levels in patients with Crohn’s disease. These findings indicate that, despite the potential diagnostic utility of periostin and galectin-3, their serum profiles do not accurately reflect disease activity in either UC or CD. Therefore, these biomarkers may be more suitable for early IBD diagnoses rather than monitoring disease activity. It should be taken into consideration that this study included patients with moderate and severe disease, but not those with mild disease or clinical remission. Consequently, the limited range of disease activity may have restricted our ability to detect correlations between the analyzed biomarkers with disease activity indices. It is therefore possible that these biomarkers could distinguish major differences in disease activity, such as between severe disease and remission, but not more subtle transitions, such as between moderate and severe disease. Therefore, future studies should include patients representing the full spectrum of disease activity—from severe disease to clinical remission—to provide a more comprehensive evaluation. Considering an above-mentioned significant correlation, galectin-3 may play a potential role in assessing the activity of the inflammatory process in CD. As previously described, galectin-3 is considered to be a key modulator of immune response. In fact, Chen et al. [34] demonstrated that galectin-3 regulates the synthesis of proinflammatory cytokines in monocyte-derived dendritic cells. Using galectin-3-specific siRNA, the researchers evaluated the effects of galectin-3 downregulation on cytokine profiles and subsequent polarization of CD4+ T cells. Galectin-3 siRNA suppressed the expression of pro-inflammatory IL-6, IL-1β and IL-23p19, along with an increase in anti-inflammatory IL-10 expression. Moreover, galectin-3 siRNA enhanced the synthesis of IFN-γ, while suppressing IL-17A and IL-5 production in CD4+ T cells. These results indicate that downregulation of galectin-3 may shift the polarization of CD4+ T cells to Th1 rather than Th17 or Th2 phenotypes. Thus, during IBD, upregulation of galectin-3 may contribute to enhanced synthesis of proinflammatory cytokines in dendritic cells and may also stimulate the activation of Th17 and Th2 cells, which are considered key pathogenic cells in CD and UC, respectively.

Moreover, in this study serum profiles of periostin, galectin-3 and tenascin C were evaluated in IBD patients before and after one year of treatment. In UC group patients received biological anti-TNF-α therapy, while CD patients were provided with conventional anti-inflammatory treatment. To the best of our knowledge, this is the first study to evaluate the effect of anti-TNF-α treatment on serum galectin-3 levels in UC patients. The implementation of biological treatment significantly increased galectin-3 serum concentrations, suggesting a potential regulatory effect of TNF-α on galectin-3 expression in UC patients. Interestingly, implemented biological treatment did not influence CRP concentrations despite CRP, being one of the most common indicators of inflammatory response and a standard tool for monitoring IBD activity. Although CRP is a routine laboratory test used for both the diagnosis and monitoring of IBD, its level is not consistently elevated in patients with active endoscopic inflammation. Our observations reinforce the notion that CRP, while widely used in routine clinical practice, is not always a reliable surrogate for mucosal inflammation in IBD [35,36,37,38,39]. Taking into consideration that in some IBD patients CRP levels remain within the normal range despite ongoing intestinal inflammation, galectin-3 measurements may offer greater benefit in evaluating proper response to biological treatment in IBD. Since no previous studies have examined the impact of anti-TNF-α therapy on galectin-3 level in UC, our results cannot be directly compared to existing data. A possible explanation for the observed increase in galectin-3 upon anti-TNF-α treatment can be drawn from the study by Qiu et al. [40], which investigated the interplay between TNF-α and galectin-3 during inflammation in keratinocytes. The researchers demonstrated that TNF-α may suppress galectin-3 expression via NF-κB signaling pathway. Interestingly this inhibitory effect was not mediated by MAPK, ERK or JNK pathways. Qiu et al. further proposed that this downregulation of galectin-3 may be mediated by TNF-α-induced expression of hsa-miR-27a-3p, however only under inflammatory conditions. Therefore, in the context of biological treatment, the suppression of TNF-α could result in reduced hsa-miR-27a-3p activity. This reduction could relieve the inhibition of galectin-3 synthesis mediated by NF-κB, leading to further increase in serum galectin-3 level in UC patients. It should be noted, however, that the suppressive role of hsa-miR-27a-3p on galectin-3 expression has been documented only in keratinocytes. Since no evidence currently exists regarding a similar mechanism in intestinal epithelial or immune cells during IBD, this hypothesis requires further experimental validation. Nonetheless, our study suggests that galectin-3 measurements may be helpful in monitoring the response to biological treatment during UC.

In contrast to post-treatment UC patients, those in the CD group exhibited significantly decreased serum galectin-3 concentration after a year of conventional anti-inflammatory treatment reaching levels comparable to those observed in healthy individuals. Considering that baseline galectin-3 levels did not differ significantly between UC and CD patients, it is possible that circulating galectin-3 concentrations were not primarily driven by disease-specific immunopathological distinctions. Instead, the contrasting trends in galectin-3 levels following treatment appear more likely to reflect treatment-specific effects and resulting molecular responses. Importantly, both biological therapy in the UC group and conventional anti-inflammatory treatment in the CD group were initiated at the beginning of this study and continued for one year, ensuring no difference in therapy duration between UC and CD groups. Therefore, the observed differences between the UC and CD groups may result from the distinct therapeutic strategies applied in each cohort. Indeed, the study by Dabelic et al. [41] demonstrated that LPS induces galectin-3 expression in monocyte-like THP-1 cells, which effect was suppressed by corticosteroid treatment. Notably, this suppression was not observed in the case of non-steroidal anti-inflammatory drugs. Moreover, the authors reported that corticosteroids modulate galectin-3 expression not only at the transcriptional level, but also at the protein level by modulating its secretion and degradation. These findings may help explain the post-treatment reduction in galectin-3 levels observed in CD patients in our study and suggest a potential role for galectin-3 as a biomarker for monitoring the efficacy of corticosteroid treatment in IBD. It should be taken into consideration that the inclusion criteria for biological therapy in UC patients required prior unsuccessful corticosteroids and immunosuppressive (azathioprine or 6-mercaptopurine) therapies, whereas in the CD group, patients received conventional anti-inflammatory treatment for the first time. As there was no significant difference in baseline galectin-3 levels between the UC and CD groups, the failure of conventional anti-inflammatory treatment in UC was not reflected by changes in galectin-3 levels. Therefore, it is possible that decrease in galectin-3 concentrations during conventional anti-inflammatory treatment may serve as a prognostic indicator of a favorable therapeutic response. Additionally, as serum CRP levels were found to correlate with galectin-3 levels in the CD group, normalization of galectin-3 following treatment may further indicate a beneficial effect of conventional treatment on ongoing intestinal inflammation. In the CD group of patients, similarly to UC, CRP levels did not change following the implemented treatment, whereas galectin-3 concentrations decreased significantly. Thus, galectin-3 may serve as a more sensitive biomarker than CRP for evaluating treatment response in CD, particularly in patients receiving conventional anti-inflammatory therapies. These results further support the potential use of galectin-3 measurements in evaluating disease activity and monitoring therapeutic response in CD.

Following treatment in IBD patients, a significant decrease in periostin levels was observed in the CD group after corticosteroid treatment. In contrast, no such change was noted in the UC group receiving anti-TNF-α biological therapy. This difference may be related to the regulatory role of IL-4 and IL-13 in periostin expression, as described in the study by Takayama et al. [42]. The researchers demonstrated that IL-4 and IL-13 activate the STAT6 signaling pathway, leading to periostin synthesis in fibroblasts. Considering that corticosteroids are known to suppress the expression of various cytokines including IL-4 and IL-13 [43,44], their inhibition during anti-inflammatory therapy might contribute to the reduced periostin synthesis observed in CD patients. The lack of change in periostin levels in UC patients may be attributed to the different therapeutical strategy, i.e., anti-TNF-α biological treatment. Indeed, Pittoni et al. [45] reported that anti-TNFα treatment did not affect IL-13 expression in patients with rheumatoid arthritis. Therefore, in UC patients, biological treatment with adalimumab may also have no impact on IL-13 expression. These findings indicate a possible role of periostin as a biomarker for evaluating the efficacy of conventional anti-inflammatory therapy in CD. In contrast to periostin, CRP concentration did not change in CD patients upon treatment. This observation suggests that periostin measurements may be more effective than CRP in monitoring the response to conventional anti-inflammatory treatment in CD patients.

In contrast to periostin and galectin-3 serum profiles, no significant changes in tenascin C levels were observed in both UC and CD patients. As previously discussed, although total tenascin C levels may remain unchanged, specific isoforms associated with inflammation might be differentially expressed in IBD. It is possible that anti-inflammatory treatments, both conventional and biological, affect levels of proinflammatory tenascin C variants without altering the total circulating tenascin C concentration. This hypothesis is supported by findings from Magnusson et al. [46], who measured serum concentrations of large tenascin C isoform in UC patients undergoing anti-TNF-α treatment (infliximab). A significant decrease in large tenascin C levels was observed in responders at both 2 and 14 weeks of treatment, while no such decrease occurred in non-responding UC patients. These results suggest that monitoring specific tenascin C isoforms, particularly the large variant, may provide more meaningful insight than total tenascin C measurements when evaluating response to anti-inflammatory therapies in IBD.

## 4. Materials and Methods

### 4.1. Study Population

**Figure 3:**
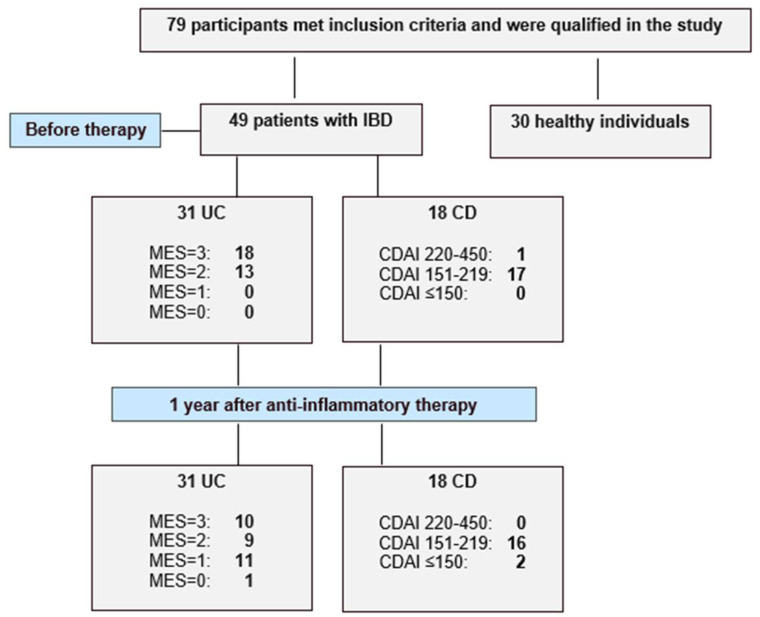
Flow chart showing the enrolled healthy individuals and active patients with ulcerative colitis (UC) and Crohn’s disease (CD), and study protocol used in the present study.

This study included 79 participants, comprising 49 patients with inflammatory bowel disease and 30 healthy individuals. A flow chart illustrating the study design and patient inclusion and exclusion is presented as Figure 3. Among the IBD patients, 31 were diagnosed with ulcerative colitis and 18 with Crohn’s disease at the Department of Gastroenterology of St. Barbara’s Regional Specialist Hospital in Sosnowiec, Poland. The diagnosis was based on endoscopic examination, clinical symptoms and laboratory tests. Disease activity was assessed using the Mayo endoscopic scale in the UC group and the Crohn’s Disease Activity Index (CDAI) for the CD group. All IBD patients were enrolled in the study according to predefined inclusion criteria, which included: age over 18 years and newly diagnosed disease (disease duration < 5 years), clinically active disease—defined as a Mayo endoscopic score ≥ 2 points in the ulcerative colitis group and a Crohn’s Disease Activity Index score ≥ 220 points in Crohn’s disease group. As patients with UC were provided with biological treatment, the inclusion criteria were consistent with those for biological therapy and additionally required documented failure of corticosteroids or immunosuppressive (azathioprine or 6-mercaptopurine) therapy. In both the UC and CD groups, patients with active bacterial, fungal or viral infections, as well as chronic liver or kidney disease, toxic or fulminant colitis or unstable coronary artery disease, were excluded. Moreover, pregnant or breastfeeding women were also not eligible for inclusion. The control group consisted of individuals over 18 years old with normal results in routine laboratory tests. Additional inclusion criteria for the control group included the absence of pharmacological treatment and no surgical procedures within one year prior to the start of the study. The exclusion criteria for participation in the control group included abnormal results in routine laboratory tests such as transaminases, bilirubin, creatinine, C-reactive protein, total protein, lipid profile and fasting glucose. Subjects with abnormal blood pressure or BMI values were also excluded from participation in our study. The biological material analyzed in this study was venous blood, collected twice from IBD patients and once from healthy individuals. For IBD patients, blood samples were taken before implementation of therapy and again after one year of treatment—biological therapy in the UC group and anti-inflammatory treatment in the CD group.

### 4.2. Methods

Serum levels of periostin, galectin-3 and tenascin C were assessed in samples obtained from IBD patients and healthy individuals. The concentration of periostin was measured with the use of ELISA kit for periostin (Cloud-Clone Corporation, Houston, TX, USA). This test was characterized by an analytical sensitivity of 33 pg/mL, and an intra-assay precision of <10%. Galectin-3 levels were determined using Galectin-3 ELISA kit from Cloud-Clone Corporation (Houston, TX, USA). The sensitivity of the test was 0.054 ng/mL with an intra-assay precision was <10%. The serum profile of tenascin C was assessed using the Tenascin C ELISA kit from Cloud-Clone Corporation (Houston, TX, USA), which demonstrated a sensitivity of 1.25 ng/mL and intra-assay error of <10%.

### 4.3. Statistical Analysis

Statistical analyses were conducted using STATISTICA software, version 13.3 (StatSoft, Cracow, Poland). The normality of data distribution was assessed using the Shapiro–Wilk test. The significance of differences in periostin, galectin-3 and tenascin C serum levels between analyzed groups was determined using Student’s *t*-test or Mann–Whitney U test, depending on the data distribution. To further evaluate the diagnostic potential of these biomarkers in ulcerative colitis (UC) and Crohn’s disease (CD), receiver operating characteristic (ROC) curve analysis was performed. Correlations between serum biomarker levels, disease activity, and C-reactive protein levels were assessed using Pearson’s or Spearman’s correlation coefficients, as appropriate based on data normality. A *p*-value of <0.05 was considered statistically significant.

The disease activity was assessed using the Mayo endoscopic scale (MES) in the UC group and the Crohn’s Disease Activity Index (CDAI) for the CD group. IBD, inflammatory bowel diseases.

## 5. Conclusions

The aim of this pilot study was to evaluate the utility of serum periostin, galectin-3 and tenascin C measurements in the diagnosis and monitoring of IBD. Compared to healthy individuals, patients with IBD demonstrated significantly decreased periostin levels and increased galectin-3 concentrations, whereas no significant differences were observed in tenascin C serum profiles. Among the analyzed biomarkers, periostin and galectin-3 presented the highest clinical utility for both UC and CD. Serum periostin profiles effectively differentiated both UC and CD patients from healthy individuals with encouraging sensitivity and negative predictive value, indicating a low risk of false negative diagnoses. Galectin-3 measurements also demonstrated high diagnostic value, albeit slightly lower than periostin. Importantly, galectin-3 levels correlated significantly with CRP concentrations, indicating its potential as a biomarker supporting the evaluation of disease activity, particularly in relation to inflammation severity. Furthermore, the implemented treatments influenced the serum levels of both biomarkers—periostin levels decreased in response to conventional anti-inflammatory treatment in CD group, whereas galectin-3 levels were modulated by both biological therapy in UC patients and conventional anti-inflammatory therapy in CD patients. Taken together, these results provide preliminary evidence that serum measurements of periostin and galectin-3 may serve as an adjunct to routine diagnostic methods, potentially improving the sensitivity and specificity not only IBD diagnosis, but also disease activity monitoring and assessment of therapeutic response. Given the limited sample size and suggested role of these biomarkers in different diseases, the findings should be interpreted with caution and validated in larger, independent studies before clinical implementation can be considered.

## Figures and Tables

**Table 1:** Clinical characteristics of patients with IBD.

Parameter		Total (N = 49)		UC (N = 31)			CD (N = 18)		
		Before Treatment	After Treatment	UC0	UC1	*p*	CD0	CD1	*p*
Females, n (%)		21 (42.8%)		13 (41.9%)		-	8 (44.4%)		-
Males, n (%)		28 (57.1%)		18 (58.1%)		-	10 (55.6%)		-
Implemented treatment		Biological or conventional anti-inflammatory treatment		Biological, TNF-α inhibitors (adalimumab)		-	Conventional anti-inflammatory, steroids and immunomodulators (azathioprine or mercaptiopurine)		-
Age [years]		32.7 ± 12	-	33 ±13	-	-	32 ± 9.6		-
BMI [kg/m^2^]		23.2 ± 3.5	22.8 ± 3.3	24.3 ± 3.6	24.5 ± 4.2	>0.05	20.6 ± 3.4	19.8 ± 2.8	>0.05
Disease activity, n (%)	Severe	35 (71.4%)	26 (53.1%)	18 (58.1%)	10 (32.3%)	-	17 (94.4%)	16 (88.9%)	-
	Moderate	14 (28.6%)	11 (22.4%)	13 (41.9%)	9 (29%)		1 (5.6%)	2 (11.1%)	
	Mild	0 (0%)	11 (22.4%)	0 (0%)	11 (35.5%)		0 (0%)	0 (0%)	
	Remission	0 (0%)	1 (2%)	0 (0%)	1 (3.2%)		0 (0%)	0 (0%)	
Disease activity in Mayo or CDAI scale		-	-	Mayo: 3 (2–3)	Mayo: 2 (1–3)	<0.005	CDAI: 290.83 ± 35.53	CDAI: 270.8 ± 44.3	>0.05
CRP [mg/L]		5.5 (2.3–19.9)	5.2 (2.2–15.2)	3.37 (1.26–15.20)	2.41 (1.51–7.62)	>0.05	13.90 (3.20–31.40)	15.2 (5.3–23.4)	>0.05
Serum calprotectin [ng/mL]		2877.2 (1801.3–4754.5)	2901.7 ± 1195.8	2782.9 (1674.2–4754.5)	2708.3 ± 890.9	>0.05	3537.52 ± 1893.78	2915.3 ± 1325.9	>0.05

Results are presented as mean ± standard deviation in normally distributed data and median with interquartile range in non-normally distributed data. BMI, body mass index; CD0, patients with Crohn’s disease before treatment; CD1, patients with Crohn’s disease after treatment; CDAI, Crohn’s disease activity index; CRP, C-reactive protein; N, number of patients; UC0, patients with ulcerative colitis before treatment; UC1, patients with ulcerative colitis after treatment.

**Table 2:** Serum profile of periostin, galectin-3 and tenascin C in IBD patients and healthy individuals.

Parametr	UC			CD			Control
	UC0	UC1	*p* UC0 vs. C	CD0	CD1	*p* CD0 vs. C	
Periostin [μg/mL]	119.33 (77.83–167.85)	99.45 (63.06–161.86)	<0.0001	98.03 ± 61.62	41.38 ± 25.19	<0.0001	548.8 ± 307.46
Galectin-3 [ng/mL]	1.63 (1.14–3.09)	2.42 (1.6–3.42)	<0.0005	1.92 ± 0.97	1.33 ± 0.77	<0.05	0.95 (0.59–2.01)
Tenascin C [ng/mL]	105.17 ± 36.69	93.48 ± 38.72	>0.05	99.91 ± 22.69	93.88 ± 31.01	>0.05	95.12 ± 26.18

Results are presented as mean ± standard deviation in normally distributed data and median with interquartile range in non-normally distributed data. CD0, patients with Crohn’s disease before treatment; CD1, patients with Crohn’s disease after treatment; UC0, patients with ulcerative colitis before treatment; UC1, patients with ulcerative colitis after treatment.

**Table 3:** Diagnostic performance of serum periostin, galectin-3 and tenascin C in differentiating patients with ulcerative colitis (UC) and Crohn’s disease (CD) from healthy individuals.

Parameter	Analyzed Groups	AUC (95% CI)	Youden Index	Cut-Off	Sensitivity (%)	Specificity (%)	PPV (%)	NPV (%)
Periostin	UC	0.922 (0.85–0.995)	0.78	308.36 μg/mL	100	77.8	82.8	100
	CD	0.943 (0.88–1)	0.78	280.90 μg/mL	100	77.8	71.4	100
Galectin-3	UC	0.745 (0.616–0.875)	0.46	0.75 ng/mL	100	46.4	65.9	100
	CD	0.691 (0.506–0.876)	0.40	1.22 ng/mL	76.9	63.0	50.0	85.0
Tenascin C	UC	0.582 (0.434–0.73)	0.23	138.32 ng/mL	22.6	100	100	53.8
	CD	0.567 (0.395–0.739)	0.25	85.28 ng/mL	82.4	42.9	46.7	80.0

AUC, area under curve; C, control group, CD, patients with Crohn’s disease, NPV, negative predictive value; PPV, positive predictive value; UC, ulcerative colitis.

## Data Availability

The original contributions presented in this study are included in the article. Further inquiries can be directed to the corresponding author.
